# Interleukin 21 Controls mRNA and MicroRNA Expression in CD40-Activated Chronic Lymphocytic Leukemia Cells

**DOI:** 10.1371/journal.pone.0134706

**Published:** 2015-08-25

**Authors:** Loris De Cecco, Matteo Capaia, Simona Zupo, Giovanna Cutrona, Serena Matis, Antonella Brizzolara, Anna Maria Orengo, Michela Croce, Edoardo Marchesi, Manlio Ferrarini, Silvana Canevari, Silvano Ferrini

**Affiliations:** 1 Functional Genomics and Bioinformatics, Department of Experimental Oncology and Molecular Medicine, Fondazione IRCCS Istituto Nazionale dei Tumori, Milan, Italy; 2 Laboratory of Biotherapy, IRCCS-AOU San Martino-IST, Istituto Nazionale per la Ricerca sul Cancro, Genoa, Italy; 3 Laboratory of Molecular Diagnostics, IRCCS-AOU San Martino-IST, Istituto Nazionale per la Ricerca sul Cancro, Genoa, Italy; 4 Scientific Direction, IRCCS-AOU San Martino-IST, Istituto Nazionale per la Ricerca sul Cancro, Genoa, Italy; University of Thessaly, Faculty of Medicine, GREECE

## Abstract

Several factors support CLL cell survival in the microenvironment. Under different experimental conditions, IL21 can either induce apoptosis or promote CLL cell survival. To investigate mechanisms involved in the effects of IL21, we studied the ability of IL21 to modulate gene and miRNA expressions in CD40-activated CLL cells. IL21 was a major regulator of chemokine production in CLL cells and it modulated the expression of genes involved in cell movement, metabolism, survival and apoptosis. In particular, IL21 down-regulated the expression of the chemokine genes *CCL4*, *CCL3*, *CCL3L1*, *CCL17*, and *CCL2*, while it up-regulated the Th1-related *CXCL9* and *CXCL10*. In addition, IL21 down-regulated the expression of genes encoding signaling molecules, such as *CD40*, *DDR1* and *PIK3CD*. IL21 modulated a similar set of genes in CLL and normal B-cells (e.g. chemokine genes), whereas other genes, including *MYC*, *TNF*, *E2F1*, *EGR2* and *GAS-6*, were regulated only in CLL cells. An integrated analysis of the miRNome and gene expression indicated that several miRNAs were under IL21 control and these could, in turn, influence the expression of potential target genes. We focused on hsa-miR-663b predicted to down-regulate several relevant genes. Transfection of hsa-miR-663b or its specific antagonist showed that this miRNA regulated *CCL17*, *DDR1*, *PIK3CD* and *CD40* gene expression. Our data indicated that IL21 modulates the expression of genes mediating the crosstalk between CLL cells and their microenvironment and miRNAs may take part in this process.

## Introduction

B-cell chronic lymphocytic leukemia (CLL) is a common type of leukemia, characterized by the progressive accumulation of CD5+ monoclonal B lymphocytes in peripheral blood, bone marrow and lymphoid tissues [1,2]. The expansion of the CLL clone is due to an imbalance between cell death and proliferation [3]. Clonal expansion occurs in specific niches within the lymphoid tissues and the bone marrow where CLL cells are protected from apoptosis [4,5]. In this supportive microenvironment, CLL cells establish interactions with multiple cell types, including activated CD4+ T cells expressing CD40 ligand (CD40L) [6]. In addition, antigenic stimulation is involved in CLL cell activation and proliferation via the triggering of their B-cell receptor (BCR) complex, and evidence from several studies indicate that CLL cells derive from antigen-experienced B-cells [7–9]. Besides CD40L and the antigen, several other molecules regulate CLL survival and proliferation. For example, nurse-like cells and stromal endothelial cells support the survival of CLL cells *in vitro* through contact-dependent stimuli, mediated by members of the tumor necrosis factor (TNF) superfamily [10,11]. In addition, several chemokines and cytokines have been reported to regulate CLL cell survival and proliferation [5]. For example, the chemokine CXC ligand 12 (CXCL12; also known as stromal cell-derived factor-1, SDF-1), which is produced by nurse-like cells [12], mediates anti-apoptotic effects in CLL cells via the CXC chemokine receptor type 4 (CXCR4). Importantly, chemokines have also been involved in orchestrating the crosstalk between CLL cells and their supportive cells within the microenvironment. Thus, CC ligand 3 (CCL3) and CCL4 are produced by CLL cells undergoing BCR stimulation or co-culture with nurse-like cells [13]. In turn, these factors attract CC receptor type 1 (CCR1)-expressing monocytes/macrophages, which activate endothelial cells to support CLL cell survival [14]. In addition, CLL cells produce CCL22 and CCL17 in response to CD40L stimulation and CCL22 attracts CCR4+CD4+CD40L+ T cells, which further stimulate CLL cells [15].

Among the cytokines, hepatocyte growth factor (HGF), which is produced by different types of mesenchymal cells, supports CLL cell survival [16]. In addition, cytokines of the interleukin (IL) 2 family, such as IL4 and IL15, mediate CLL cell survival and proliferation [17,18]. In contrast, IL21, a regulator of normal B-cell survival [19] and differentiation, [20] was shown to induce CLL B-cell apoptosis [21–23]. These data were obtained using recombinant IL21 at pharmacological dosages (50–100 ng/ml). However, IL21 produced by CD4+CD40L+ T cells supported the proliferation of co-cultured CLL cells [24], acting in concert with other T-follicular helper-derived cytokines such as IL4 [25]. Another report suggested that IL21, in combination with toll-like receptor (TLR) 9 agonists, exerts differential effects on CLL cells from progressing or non-progressing patients [26]. IL21 may also induce CLL B-cell differentiation through the induction of B-lymphocyte-induced maturation protein-1 (Blimp-1), a regulator of plasma cell induction [27].

Moreover, IL21 also mediates the apoptosis of various non-Hodgkin’s lymphomas, including follicular [28], mantle cell [29] and diffuse large B-cell lymphoma [30]. The observation that IL21 inhibits the survival of some neoplastic B-cells may have translational implications. Indeed, a phase I dose-finding trial of IL21 and rituximab in relapsed and refractory low-grade B-cell malignancies suggested clinical activity [31]. Several lines of evidence indicate that IL21-mediated effects require gene transcription through the JAK3/STAT-1 and -3 pathways, which mediate apoptosis in sensitive cells [19,21–23,28–30]. Other cytokines, such as interferons, may regulate gene expression through the modulation of specific miRNAs, which target specific mRNAs [32,33].

To gain additional knowledge on the molecular mechanisms of IL21 activity on neoplastic B-cells, we studied the ability of IL21 to modulate gene and miRNA expressions in CD40-activated CLL cells. An integrated analysis of miRNA and mRNA expression suggested a role of miRNAs in the regulation of gene expression by IL21.

## Materials and Methods

### CLL and normal B-cell isolation, activation and apoptosis detection

This study was approved by the institutional review board of Istituto Nazionale per la Ricerca sul Cancro (Genoa, Italy) and by the Ethical Committee of Regione Liguria. Peripheral blood samples were obtained from untreated CLL patients (Table 1) upon written informed consent, according to our institutional procedure and the Declaration of Helsinki. B-cell populations were enriched from peripheral blood mononuclear cells by negative selection with antibody-coated magnetic beads (anti-CD2; Dynal-Invitrogen, Oslo, Norway) to consist of >95% of CD19+/CD5+ cells. The B-cell RosetteSep kit (Stemcell Technologies, Grenoble, France) was used to isolate normal B-cells from buffy coats of six age-matched healthy donors. CLL or normal B-cells were then pre-activated by co-culture with adherent, irradiated CD40L-expressing murine fibroblasts for 36–48 h, collected and stimulated for an additional 18 h with either medium only or recombinant human IL21 produced in *E*. *coli* (Biosource-Invitrogen, San Diego, Ca) at 80 ng/ml as described [21]. The CD40L-expressing NIH-3T3 murine fibroblast cell line, stably transfected with human CD40L cDNA, was kindly provided by Franco Fais (Genova, Italy) and was generated as previously described [34]. A dose of 80 ng/ml was used, based on dose—response analyses. CD40L pre-stimulation allowed the enhancement of IL21 receptor (IL21R) expression on CLL cells and their sensitization to IL21 activity [21]. Apoptosis was assessed by fluorescence-activated cell sorting (FACS) analysis of annexin V—fluorescein isothiocyanate (FITC) and propidium iodide (PI) staining (Bender, Vienna, Austria).

**Table 1:** Prognostic markers of CLL cases and assays performed.

Patient	CD38%	ZAP70%	IgVH Mutations	Assays
1	75.5	90	Unmutated	MA, RT-qPCR, miR
2	0.8	16	Mutated	MA, RT-qPCR
3	50	10	Mutated	MA, RT-qPCR
4	17	31	Unmutated	MA, RT-qPCR
5	20	20	Mutated	MA, RT-qPCR
6	53	24	Mutated	MA, RT-qPCR, miR
7	82	67	Unmutated	MA, RT-qPCR, miR
8	7	35	Mutated	MA, RT-qPCR, miR
9	82	47	Unmutated	MA, RT-qPCR, miR
10	35	42.5	Unmutated	MA, RT-qPCR, miR
11	2	19	ND	MA, RT-qPCR, miR
12	1.9	1	Mutated	MA, RT-qPCR, miR
13	99	6.7	Unmutated	MA, RT-qPCR, miR
14	20.5	47	Unmutated	ELISA, RT-qPCR
15	nd	59	Unmutated	ELISA, RT-qPCR
16	28	59	Unmutated	ELISA, RT-qPCR
17	0.5	39	Mutated	ELISA, RT-qPCR
18	0.8	33.5	Mutated	ELISA, RT-qPCR
19	nd	Nd	Mutated	ELISA, RT-qPCR
20	1	4	Mutated	ELISA, RT-qPCR
21	1	5	Mutated	ELISA, RT-qPCR
22	2.5	29.2	Mutated	ELISA, RT-qPCR
23	99.9	30	Unmutated	ELISA, RT-qPCR
24	12.5	56.3	Mutated	ELISA, RT-qPCR
25	14	30	Mutated	ELISA, RT-qPCR, TR
26	nd	nd	Mutated	ELISA, RT-qPCR, TR
27	42.6	47.1	Unmutated	RT-qPCR, TR
28	40.5	49.5	Unmutated	RT-qPCR, TR
29	10	50	ND	RT-qPCR, TR

All patients were untreated and at diagnosis. ND = not determined; MA = microarray for gene expression profiling (training set); miR = analysis of miRNome; ELISA = validation for chemokines by ELISA; TR = miR/antagomiR transfection assay.

### Microarray analysis

Total RNA was isolated from both untreated and IL21-stimulated CLL and normal B cells, using TRIzol (Invitrogen-LifeTechnologies, Carlsbad, CA, USA). Total RNA from 13 paired untreated and IL21-stimulated cells was then amplified *in vitro*, labeled with biotin and hybridized on Sentrix Bead Chip HumanRef_8_v2 (Illumina, San Diego, CA, USA). Array chips were washed, stained with 1 mg/ml of Cy3-streptavidine (GE Healthcare, Pittsburgh, PA) and scanned with Illumina BeadArray Reader (Illumina, San Diego, CA, USA). For each gene, a detection threshold with a *p* value < 0.05 was set, and 50% of missing values were allowed as a cutoff to filter the reliable data, yielding an expression matrix containing 12,166 probes. In addition, nine pairs of samples were miRNA-profiled on Illumina Human miRNA_v2 chips. Mature miRNAs were amplified, fluorescently labeled, hybridized on Illumina miRNA BeadChips and analyzed by the Illumina BeadArray Reader, as described previously [35]. Primary data were collected using BeadStudio V3.0 software (Illumina, San Diego, CA, USA). All microarray data were compliant to MIAME (Minimum Information About a Microarray Experiment) guidelines and were deposited into the Gene Expression Omnibus (GEO) database of NCBI (National Center for Biotechnology Expression) (http://www.ncbi.nlm.nih.gov/geo/), with accession numbers GSE42158 and GSE42160.

### Quantitative reverse transcriptase polymerase chain reaction (RT-qPCR) analysis

TaqMan gene primer sets were purchased from Life Technologies (LifeTechnologies, Carlsbad, CA, USA). Reverse transcription and PCR amplification for gene expression assays were performed with TaqMan Transcription and TaqMan Gene Expression Master Mix, respectively, following the manufacturer’s instructions (LifeTechnologies, Carlsbad, CA, USA). RT-qPCR data were normalized, using POL2RA as housekeeping gene.

miRNA RT-qPCR was performed using the miRCURY LNA Universal RT microRNA PCR system (Exiqon, Vedbaek, Denmark) according to the manufacturer’s instructions.

Total RNA (20 ng) was polyadenylated and reverse-transcribed at 42°C (60 min), in a reaction volume of 20 μl using a poly-T primer containing a 5′ universal tag. After heat-inactivation at 85°C the resulting cDNA was diluted 80-fold in nuclease free water, and a volume of 8 μl was amplified in 20 μl reaction volume as follows: 95°C for 10 min, followed by 40 cycles at 95°C for 10 s and 60°C for 60 s. Normalization was performed with SNORD48 [36].

Gene and miRNA expression levels were quantified, using a sequence detection system (ABI Prism 7900HT; LifeTechnologies, Carlsbad, CA), and the threshold cycle (Ct) for each sample was determined. ABI SDS 2.4 software (LifeTechnologies, Carlsbad, CA, USA) was used to recover the data, and the relative expression was calculated, using the comparative ΔCt method.

### Enzyme-linked immunosorbent assay (ELISA) for chemokines

Supernatants from either untreated or IL21-treated CLL cells were harvested 36 h post stimulation and assayed by ELISA for CCL3, CCL4, CCL17, CCL22, CXCL9 and CXCL10 according to the manufacturer’s protocol (R&D Systems, Minneapolis, MN, USA).

### miRNA and antagomir transfection

Hsa-miR-663b, its specific antagonist or an irrelevant synthetic RNA (Qiagen, Milan, Italy) were transfected, in triplicate, in CD40-activated CLL cells (10^6^ cells) at 50 nmol/l, using the AMAXA nucleofection system, the human B-cell kit and the U-015 program (Lonza, Cologne, Germany). After 18 h of culture, RNA was extracted for mRNA analysis.

### Statistics and bioinformatic analyses

Differentially expressed genes were defined according to the following criteria: false discovery rate (FDR) <0.01 and a fold-change threshold >1.5 or < 0.667. The open-source MultiExperiment Viewer v4.6 was used for principal component analysis (PCA), and the BrB ArrayTools_v4.1.0-stable release (http://linus.nci.nih.gov/brb) was used for class comparison, hierarchical clustering, global test and heat map analyses. In this study, we used weighted gene co-expression network analysis (WGCNA) [37] to determine the gene co-expression networks regulated by IL21 in CD40-activated CLL cells from microarray gene expression data. WGCNA is a systems biology method that enables the description of the correlation patterns among genes and the identification of modules of highly correlated genes across microarray samples. As a result, WGCNA offers a meaningful data reduction scheme, focusing the analysis on modules expected to correspond to specific biological functions. The method is based on the computation of pair-wise Pearson correlations between each gene pair, allowing the assessment of the topological overlap (TO), a measurement of the relationships of the two genes against all other genes. A hierarchical map, using 1-TO as the distance measure, was built to cluster the genes, and a tree-cutting algorithm was used for module assignments. The module eigengene (ME) is a representative value of each module, which is equivalent to the first principal component and explains the largest proportion of variance of the module genes. Submap analysis was performed, using subclass mapping [38], to assess the degree of molecular correspondence among different datasets. IPA 8.5 (Ingenuity Pathway Analysis, Ingenuity Systems; Qiagen Venlo, The Netherlands) was used to analyze the functional and network connections. Integration of miRNA and mRNA data was performed, using the MAGIA tool [39], and visualized by Cytoscape [40] miRNA, gene expression and protein levels were compared, using a two-sample paired Student's *t*-test with random variance model.

## Results

### IL21 regulated gene expression in CD40-activated CLL cells

**Fig 1:**
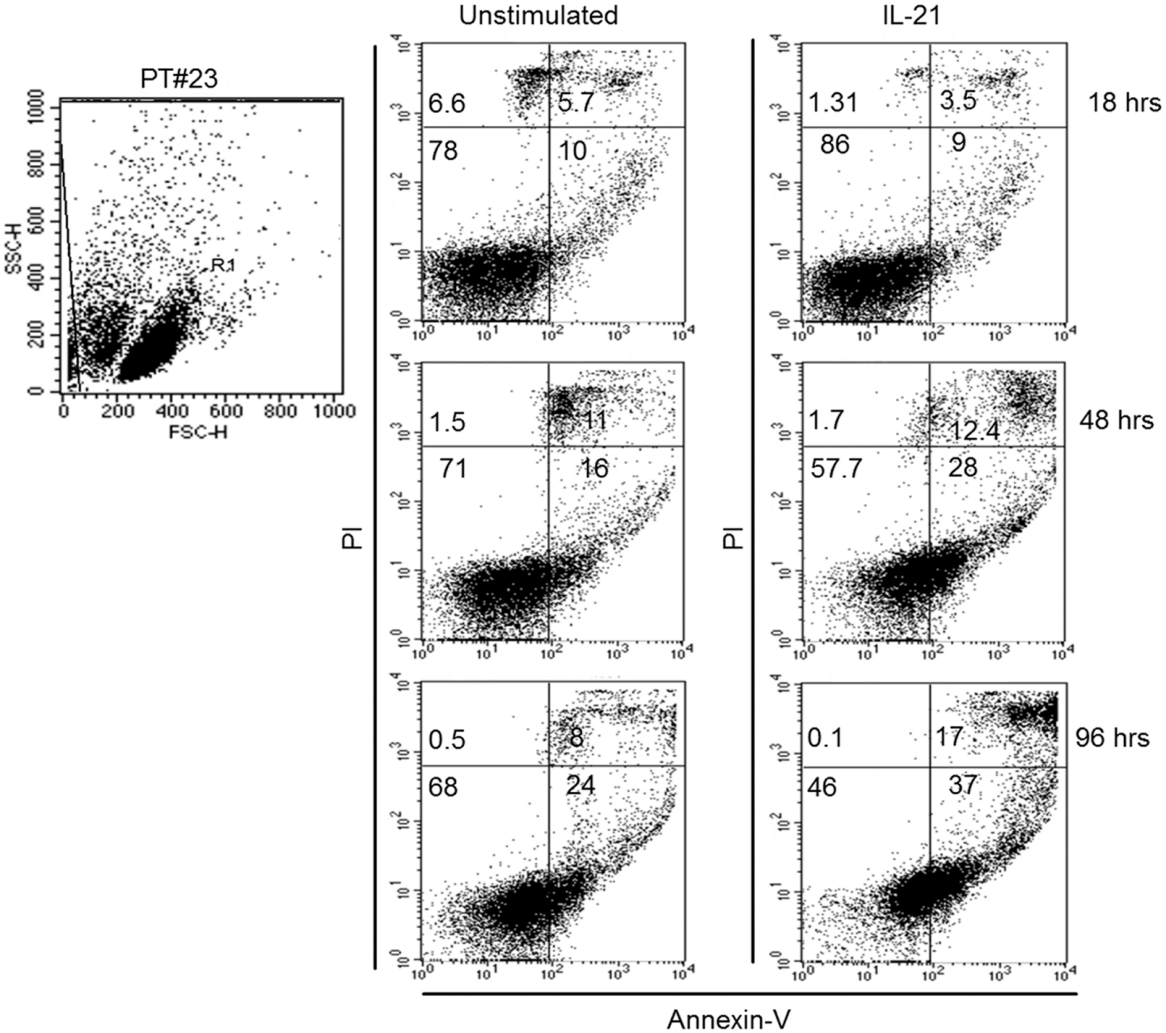
Time-course of IL-21 activity. IL21 (80 ng/ml) induced apoptosis of CD40-activated CLL cells only at late time points, as detected by FACS analysis of annexin V/PI staining. Gating and dot plots from a representative case are shown.

Under the experimental conditions used, IL21 induced a slow-rate apoptotic process, which became clearly evident at 48–96 h, while no significant apoptosis was induced by IL21 at 18 h when compared to untreated cells (Fig 1). IL21 induced apoptosis (>10% of induction relative to untreated cells) in 40% of cases, and no correlation between apoptosis and CLL clinical and biological characteristics was evident.

**Fig 2:**
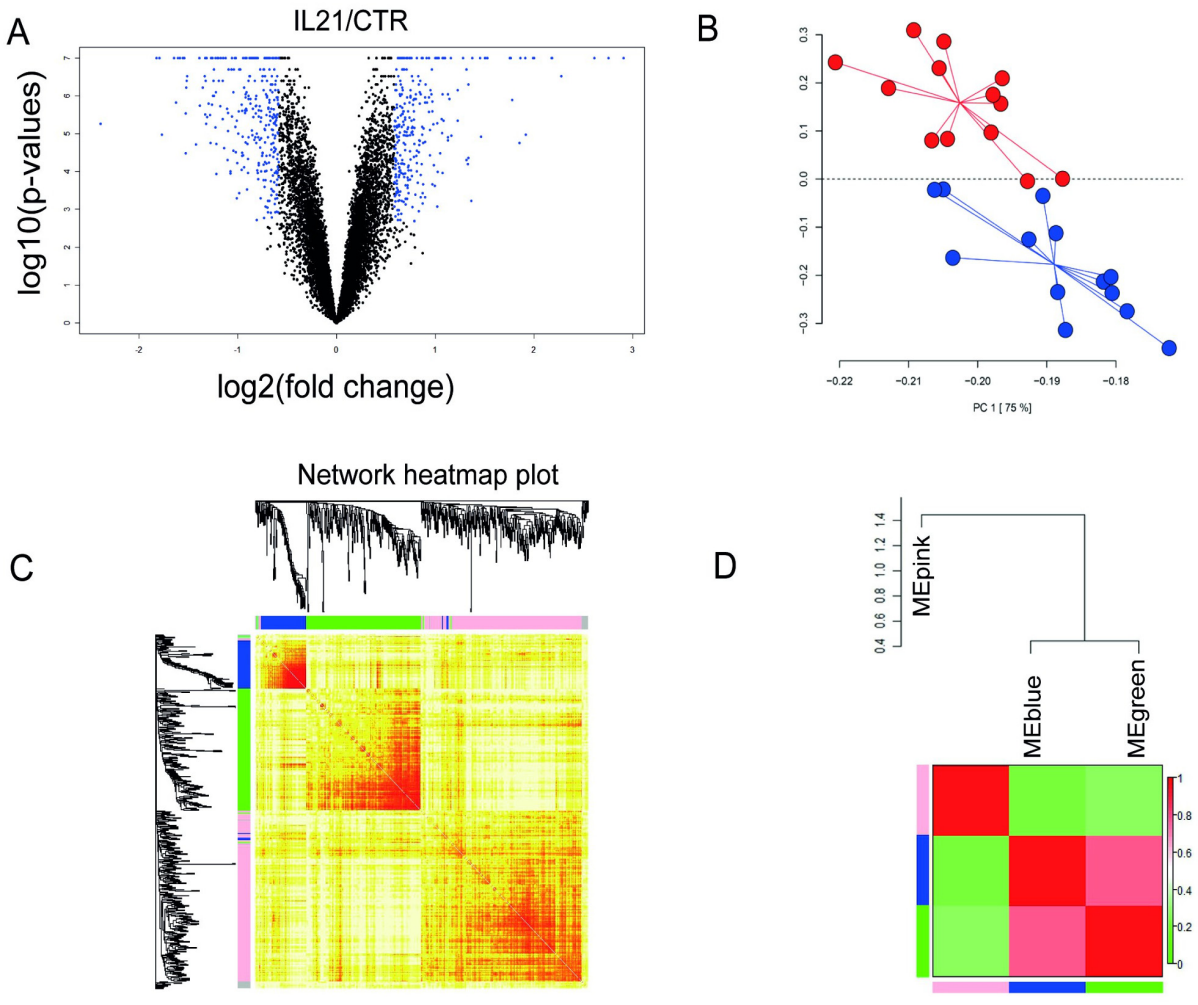
Bioinformatic analyses of IL21-modulated genes in activated CLL cells. (*a*) Volcano plot for the 12,166 genes detected by microarray analysis. The X-axis represents the fold-change, and the Y-axis represents the −log_10_ *p* value. A total of 582 probes (blue dots) were expressed differentially in IL21-treated, relative to untreated CLL cells, imposing an FDR < 0.01 and a fold-change > 1.5 or < 0.667. Global test: probability of obtaining at least 582 probes significant by chance if there were no real differences between the classes; *p* = 0.026. (*b*) The differentially expressed genes, as visualized by PCA, separated the samples in two well-defined groups corresponding to IL21-treated (blue) and untreated (red) cells. (*c*) WGCNA was performed on differentially expressed genes; clusters of like-regulated genes were referred to as modules by color (pink, green and blue). Those few genes (19) not included in the modules are in grey. In the heatmap, the intensity of red coloring indicates a high correlation of expression. (*d*) Eigengene dendogram and heatmap. Two correlated eigengenes, corresponding to MEgreen and MEblue, clustered together and differed from MEpink.

Then we studied how IL21 could modify gene expression by means of high-throughput analysis of CLL cells from a first cohort of 13 patients (Table 1). A large number of genes were modulated following IL21 treatment, as represented by the volcano plot (Fig 2A). According to the imposed thresholds, a total of 563 differentially expressed genes (corresponding to 582 probes) were retrieved, among which 275 genes were up-regulated and 288 down-regulated. PCA showed that the samples were distributed in two main clusters, matching the IL21-treated and -untreated CLL cells (Fig 2B). In this cohort, the pattern of IL-21-regulated genes was not significantly different in subgroups stratified on the basis of IgVH mutational status or of ZAP70 or CD38 expression.

Submap analysis [39] of four publicly available datasets of IL21-treated *vs*. untreated (*i*) human CLL cells (GSE50572) [24] (*ii*) human Sezary T cells (GSE8685) [41] (*iii*) murine naïve CD8+ T cells (GSE2059) [42] and (*iv*) murine pre-activated CD4+ T cells (GSE19198) [43] showed regulation of the same or orthologous genes coherent with those identified in our dataset (S1 Fig).

The dendrogram obtained by the WGCNA method showed that the differentially expressed genes clustered into three modules (defined as ME ‘color’ module), representing correlated networks of genes within each module (Fig 2C). To determine potential relationships among the modules, a set of seed “eigengenes”, one for each module, was computed. The heatmap of the three module “eigengenes” (MEs) indicated a high level of correlation between the green and blue modules and, to a lesser extent, to the pink module (Fig 2D). The genes entering into each module are listed in S1 Table along with FDR and fold-change.

Functional analyses of signaling pathways and network connections were performed by IPA. S2 Table shows the top functions (imposing a score of > 30) associated with each module.

In the largest module (MEpink module) that included 272 genes, the three top functions were: (*i*) lipid metabolism, small molecule biochemistry, hematological system development and function, (*ii*) humoral immune response, protein synthesis, cellular development and (*iii*) endocrine system disorders, gastrointestinal disease, immunological disease (S2 Table). Indeed, several modulated genes encoded cytokine receptors (*IL2RA* and *IL2RB* up-regulated; *IL13RA1* down-regulated), cytokines (*IL12A* and *TNFSF10* up-regulated; *EBI3*, *IL15*, *TNF* and *TNFSF4* down-regulated) and chemokines (*CCL17* and *CCL22* down-regulated). The three most significant molecular pathways contain TNF, nuclear factor kappa B (NF-κB) or major histocompatibility complex (MHC) class II as hubs (S2 Table).

**Fig 3:**
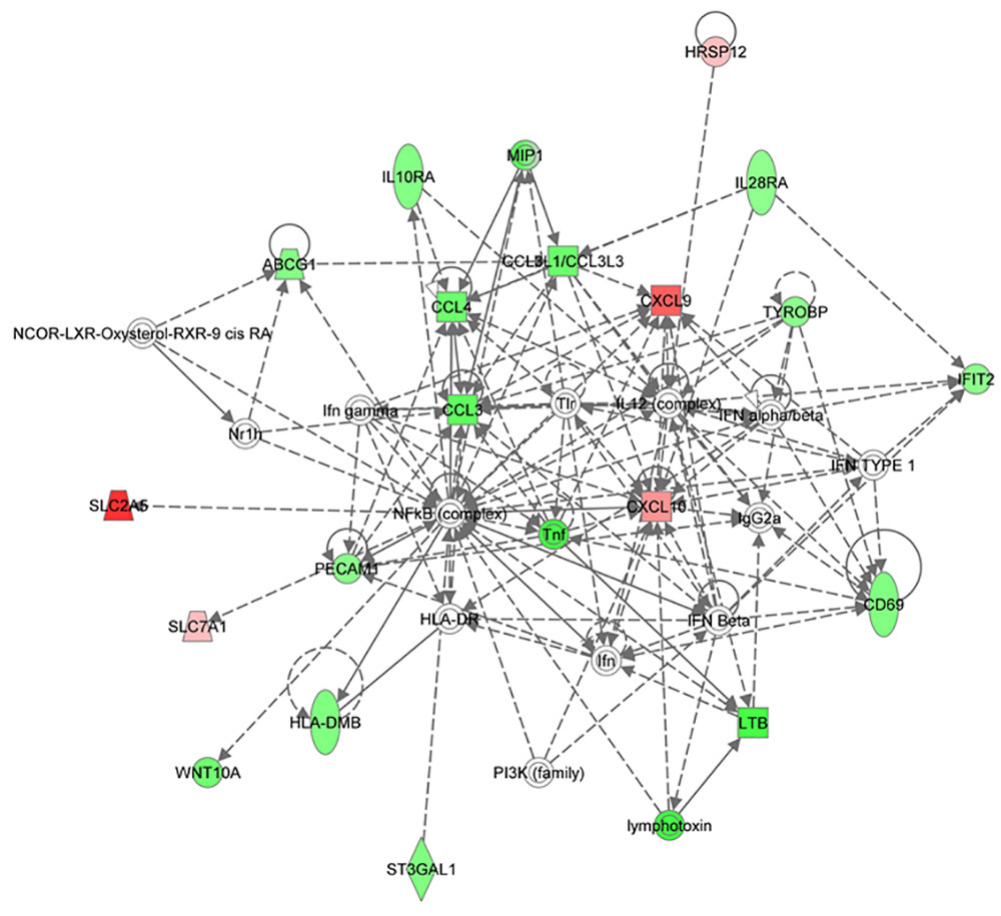
Functional network identified by IPA. IPA analysis identified a functional network in the MEgreen module, corresponding to cell-to-cell signaling and interaction, cellular movement, hematological system development and function, with NF-κB as a hub. The network is displayed graphically as nodes (genes) and edges (the biological relationships between nodes). The node color intensity is related to the IL21-mediated changes in gene expression levels (red, up-regulated; green, down-regulated genes).

In the MEgreen module that included 206 genes, the four top functions were: (*i*) RNA post-transcriptional modification, cellular assembly and organization, cellular function and maintenance, (*ii*) RNA post-transcriptional modification, infectious disease, cardiovascular disease, (*iii*) cell-to-cell signaling and interaction, cellular movement, hematological system development and function, and (*iv*) carbohydrate, lipid and nucleic acid metabolism (S2 Table). These networks suggest that IL21 may profoundly affect the metabolism of CLL cells. Networks 1 and 3 had the phosphoinositide 3 kinase (PI3K) and NF-kB complexes as respective hubs (S2 Table and Fig 3). Interestingly, network 3 included the pro-inflammatory chemokines *CCL3*, *CCL4* and *CCL3L1/LOC730422*, as well as the cytokine genes *LTB* and *TNF*, all down-regulated in IL21-treated CLL cells. In contrast, *CXCL9* and *CXCL10* chemokine genes were among the genes mostly up-regulated by IL21. In addition, the MEgreen module contained *MYC* and *MYC*-associated genes, which were all up-regulated by IL21 (S2 Table).

The MEblue module included 85 genes that were mainly up-regulated (84%) by IL21. The three top functions were: (*i*) DNA replication, recombination and repair, cell cycle, cancer, (*ii*) cancer, lipid metabolism, molecular transport, and (*iii*) cellular assembly and organization, cellular function and maintenance, post-translational modification (S2 Table). Network 1 contained the highest number of up-regulated genes, including the transcription factor *E2F1*, the MCM (minichromosome maintenance) family members *MCM-4*, *-5*, *-6*, *-7* and *-10* and the growth arrest *(GAS)-6* gene (S2 Table).

### IL21 regulated the expression of chemokine mRNA and protein

**Fig 4:**
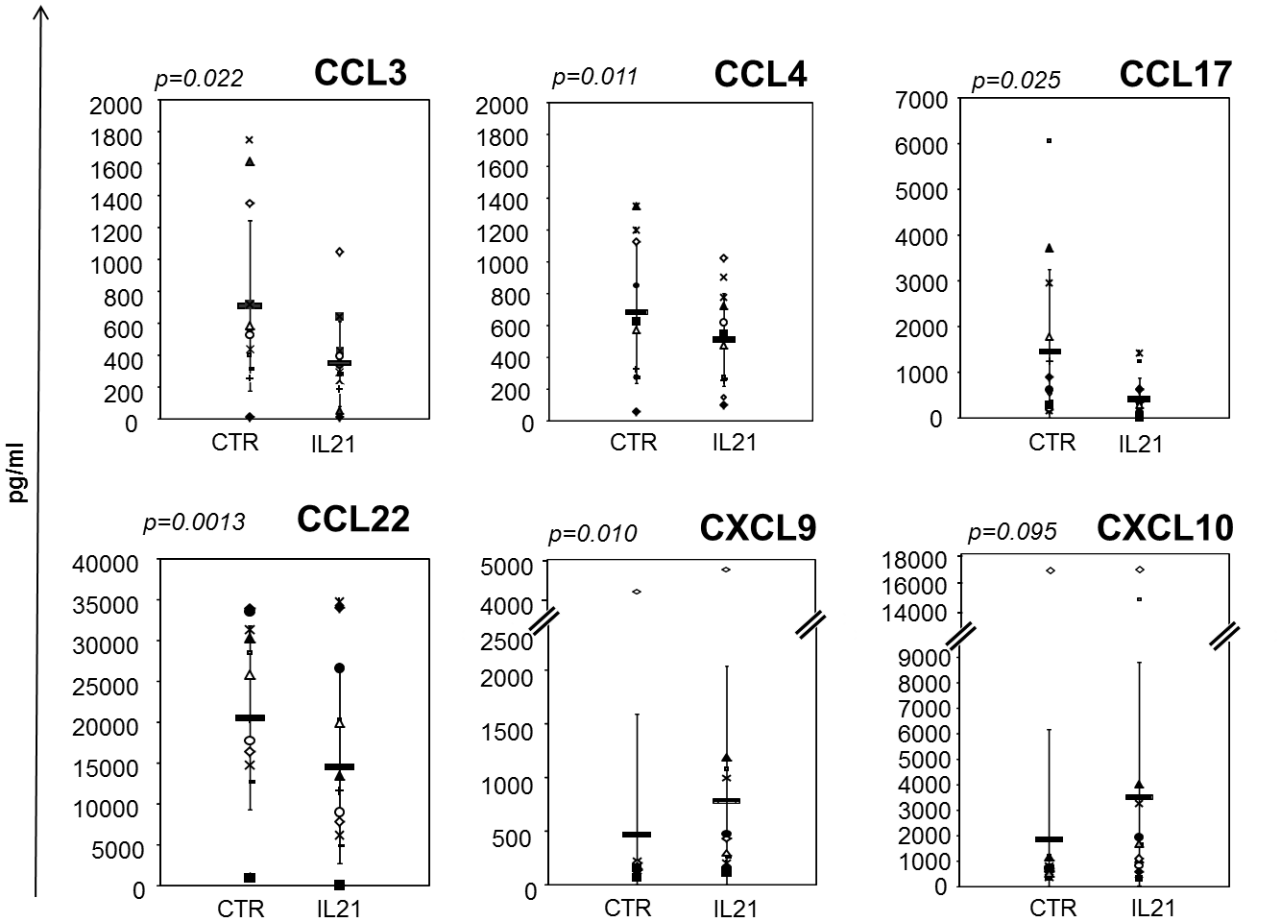
Effects of IL21 on chemokine protein levels in CLL-stimulated cells. CD40-pre-activated CLL cells were cultured for 36 h in the presence or absence of IL21, and their conditioned media were tested for chemokine concentrations by ELISA. IL21 inhibited the secretion of CCL22, CCL17, CCL4 and CCL3 from CLL cells in different patients (no. 13), while it up-regulated CXCL9 and CXCL10 secretion. *p* values were obtained using two-tailed paired *t*-test.

In view of the number of chemokine genes modulated by IL21 and their role in CLL biology,the expression of these genes was confirmed in the same cohort and validated on a distinct set of 16 CLL cases (Table 2). RT-qPCR analyses confirmed that IL21 inhibited significantly the expression of *CCL3*, *CCL3CL1*, *CCL4*, *CCL17* and *CCL22* mRNA, relative to untreated cells, while it up-regulated the Th1-related *CXCL9* and *CXCL10* chemokine gene expression. As a control, the chemokine *CCL20*, not differentially expressed in the arrays, was also assessed by RT-qPCR, confirming the absence of modulation by IL21. In addition, a group of biologically relevant genes were validated using RT-qPCR and showed consistent modulation by IL21 (Table 2). We then verified whether IL21 regulated chemokine protein production in a set of CLL cases (Table 1). The concentration of each chemokine was assessed by ELISA, in conditioned media of IL21-treated or untreated CLL cells. IL21 significantly inhibited CCL3, CCL4, CCL17 and CCL22, whereas it induced CXCL9 and CXCL10 secretion (Fig 4). Altogether, these data indicated that IL21 is a major regulator of chemokine expression in CD40-activated CLL cells.

**Table 2:** Gene-expression in IL21 treated vs untreated CLL and normal B-cells, as assessed by RT-qPCR.

Gene	Arrays^1^		Technical validation by RT-qPCR^1^		Independent validation by RT-qPCR^2^		Normal B cells by RT-qPCR^3^	
Gene symbol	Module	Fold change*	Fold change*	p-value	Fold change*	p-value	Fold change*	p-value
**CCL17**	MEpink	0.19 **↓↓**	0.08 **↓↓**	0.00019	0.1 **↓↓**	0.000136	0.14 **↓↓**	0.000538
**CCL22**	MEpink	0.35 **↓↓**	0.21 **↓↓**	0.00167	0.13 **↓↓**	0.0000026	0.36 **↓↓**	0.00226
**CD40**	MEpink	0.66 **↓**	0.53 **↓**	0.000136	0.51 **↓**	0.00812	0.5	0.118
**DDR1**	MEpink	0.44 **↓↓**	0.27 **↓↓**	3.53E-05	0.39 **↓↓**	0.000558	0.46	0.9
**EBI3**	MEpink	0.52 **↓**	0.4 **↓↓**	0.000153	0.36 **↓↓**	0.000108	0.73 **↓**	0.0114
**ITPKB**	MEpink	0.61 **↓**	0.54 **↓**	0.00606	0.74 **↓**	0.0348	0.74 **↓**	0.0348
**PIK3CD**	MEpink	0.59 **↓**	0.42 **↓↓**	0.000602	0.43 **↓↓**	0.000312	0.52 **↓**	0.00791
**TNF**	MEpink	0.61 **↓**	0.44 **↓↓**	0.0009631	0.57 **↓**	0.067	1.11	0.731
**TNFSF4**	MEpink	0.4 **↓↓**	0.34 **↓↓**	0.00903	0.5 **↓↓**	0.0013	0.37 **↓↓**	0.000215
**TSPAN33**	MEpink	0.43 **↓↓**	0.25 **↓↓**	1.84E-05	0.6 **↓**	0.00832	0.62 **↓**	0.00181
**CCL3**	MEgreen	0.46 **↓↓**	0.25 **↓↓**	0.0002371	0.56 **↓**	0.0027	0.6 **↓**	0.00625
**CCL3L1**	MEgreen	0.51 **↓**	0.11 **↓↓**	3.10E-06	0.42 **↓↓**	0.000296	0.79	0.149
**CCL4**	MEgreen	0.51 **↓**	0.14 **↓↓**	0.0004231	0.2 **↓↓**	0.0000307	0.67	0.0684
**CXCL9**	MEgreen	3.78 **↑↑**	32.5 **↑↑**	5.00E-07	2.39 **↑↑**	0.0174	10.49 **↑↑**	0.00448
**CXCL10**	MEgreen	2.53 **↑↑**	8.31 **↑↑**	0.00194	3.52 **↑↑**	0.00915	4.94 **↑↑**	0.000961
**LTB**	MEgreen	0.40 **↓↓**	0.18 **↓↓**	0.0001214	0.46 **↓↓**	0.0154	0.29 **↓↓**	0.00193
**MYC**	MEgreen	2.34 **↑↑↑**	6.77 **↑↑**	8.90E-06	4.97 **↑↑**	0.000315	1.31	0.15
**E2F1**	MEblue	1.52 **↑↑**	3.1 **↑↑**	0.000394	2.53 **↑↑**	0.00094	1.15	0.274
**EGR2**	MEblue	2.09 **↑↑**	2.36 **↑↑**	0.00203	3.18 **↑↑**	0.000003	1.01	0.78
**GAS6**	MEblue	4.55 **↑↑**	16.31 **↑↑**	1.01E-05	8.18 **↑↑**	0.00255	1.79	0.117
**CCL20**	**not DE**	**↔**	0.98	0.926	1.29	0.351	1.03	0.614

*IL21/CTR

^1^: RNA from 13 samples (patient code 1 to 13 in Table 1);

^2^: RNA from 16 samples (patient code 14 to 29 in Table 1);

^3^: RNA from 6 healthy donors

### Gene-expression IL21 regulation in CD40 activated normal B-cells

IL21 regulated a similar set of genes in normal CD40-activated B-cells isolated from the peripheral blood of age-matched healthy donors (Table 2). Most of the RT-qPCR-validated genes of the MEpink and MEgreen modules, including chemokine genes, were regulated in a similar fashion in CLL and normal B-cells. However, *TNF* and *MYC* genes, modulated in CLL cells, were unchanged in normal B-cells. Intriguingly, the three validated genes of the MEblue module *E2F1*, *EGR2* and *GAS-6* also showed no changes in normal B-cells. Thus, IL21 modulates the expression of several genes in both normal and CLL B-cells, although a set of genes is uniquely regulated in CLL cells.

### Integrated analysis of miRNome and gene expression profiling indicated a potential role of miRNA in IL21-mediated gene regulation

To investigate whether IL21 could regulate gene expressions through specific miRNAs, we analyzed whether IL21 modified the miRNome in CLL cells and performed an integrated analysis of miRNAs and gene expression profiles. Among the 565 miRNAs detected consistently in the nine sample pairs analyzed (Table 1), 63 miRNAs were modulated significantly by IL21 treatment. Thirty-nine of these miRNA were up-regulated, and 23 down-regulated, at *p* < 0.05, as shown by class comparison analysis (S3 Table). Three representative miRNAs (up-regulated hsa-miR-663b, and down-regulated hsa-miR-125b-1* and hsa-miR-708) were validated further by RT-qPCR, which confirmed the array data (S4 Table). In normal B-cells, IL21 significantly modulated hsa-miR-663b but not hsa-miR-125b-1* and hsa-miR-708 expression (S4 Table).

**Fig 5:**
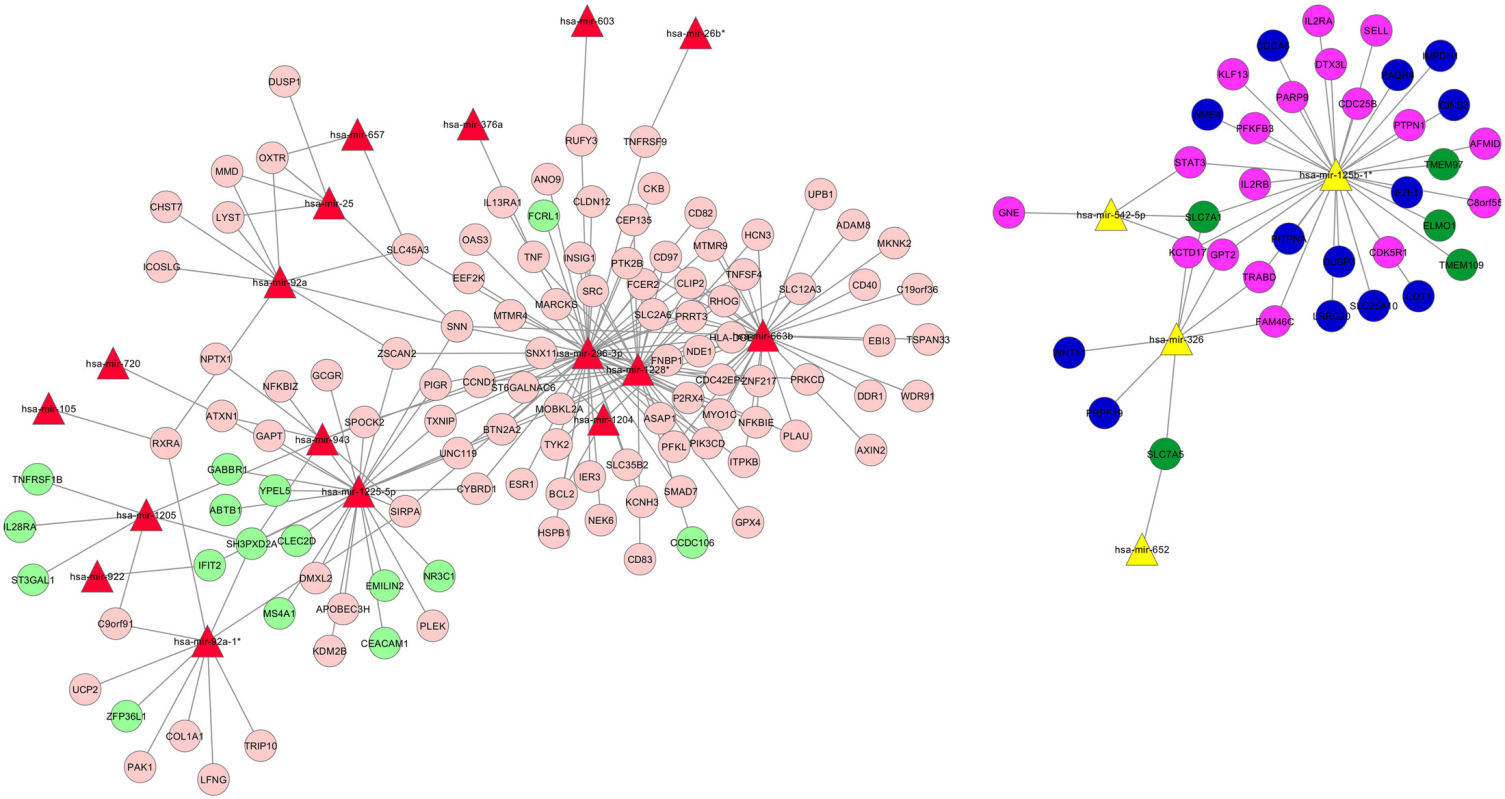
Computational integration of IL21-regulated genes and miRNAs using the MAGIA bioinformatic tool. The network of the differentially expressed miRNAs and their anti-correlated genes was computed. The top 250 interactions were used to generate the network using the Cytoscape tool. Red triangles indicate IL21-up-regulated miRNAs and yellow triangles indicate IL21-down-regulated miRNAs. The negatively correlated genes are indicated in colors referring to their related module (pink, blue and green). Light colors indicate genes down-regulated and dark colors genes up-regulated by IL21.

We compared the differentially expressed miRNAs to the differentially expressed genes in the same samples using the MAGIA software [39]. Overall, 33 miRNAs (S5 Table) had a negative correlation with 290 of the 550 genes belonging to the modules. Nine miRNAs were potentially master regulators of 73% of genes differentially expressed, and their potential targets were distributed in all three modules. Among these miRNAs, hsa-miR-296-3p, hsa-miR-125b-1*, hsa-miR-1225-5p and hsa-miR-1228* showed the largest number of potential targets (S5 Table and Fig 5). In contrast to these miRNAs, the potential target genes (27/28) of hsa-miR-663b clustered into the MEpink module (S5 Table).

The negatively correlated miRNA—mRNA interactions (S1 Table) suggested the interplay between specific IL21-regulated miRNAs and potential target genes. The MAGIA tool clustered the negatively correlated miRNA/gene interactions into two main networks (Fig 5). The largest network included 17 IL21-up-regulated miRNAs, among which hsa-miR-296-3p, hsa-miR-663b and hsa-miR-1228* showed the largest number of interconnected target genes. The other network included the IL21-down-regulated miRNAs hsa-miR-125b-1*, hsa-miR-326, hsa-miR-542-5p and hsa-miR-652 and their inversely regulated target genes.

### Role of hsa-miR-663b in IL21-mediated gene regulation

The miRNA hsa-miR-663b targeted specifically a set of genes belonging to a single module and pathway (S5 Table, S2 Fig). As this finding suggested its involvement in a functional network encompassing cellular movement and hematological system development and function, we investigated this miRNA further. In particular, we focused our attention on its potential effect on *CCL17*, *CD40* and *PI3KCD* mRNAs, which are biologically relevant to CLL, and on *DDR1* mRNA that encodes a tyrosine kinase receptor expressed in several tumors and leukemias [44]. To verify that hsa-miR-663b can affect gene expression, CD40L-activated CLL cells from five different patients were transfected with either hsa-miR-663b or an irrelevant RNA sequence. Conversely, IL21-stimulated CLL cells were transfected with either the hsa-miR-663b antagonist or the irrelevant RNA. An experimental control using RT-qPCR, showed that both hsa-miR-663b transfection and IL21 treatment strongly enhanced hsa-miR-663b intracellular levels in CLL cells (S3 Fig). Transfection with the antagonist down-regulated hsa-miR-663b levels in IL21-treated cells compared to cells transfected with an irrelevant RNA sequence (S3 Fig). This finding is in agreement with the concept that antagomir/miRNA duplexes are degraded.

**Fig 6:**
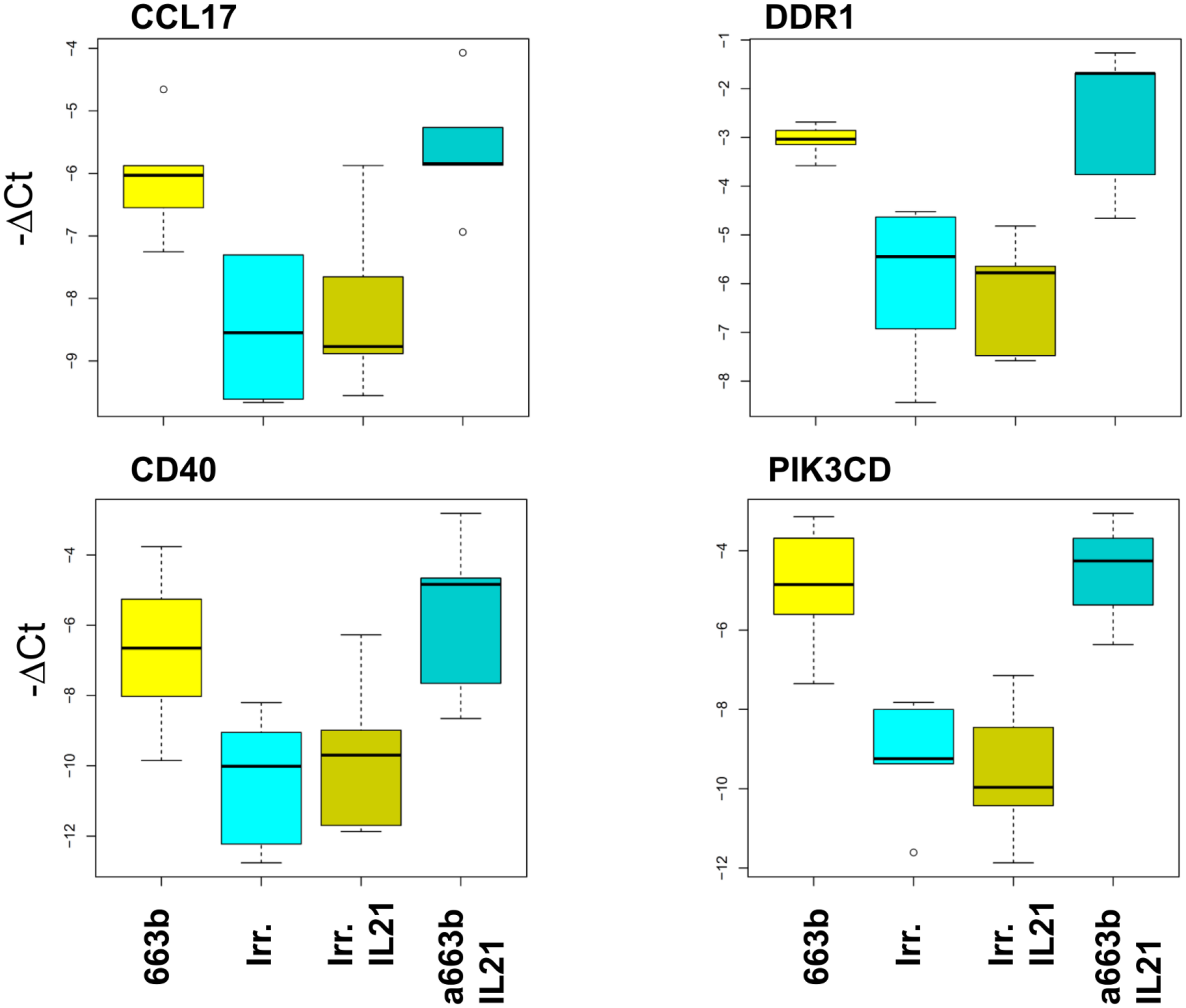
Role of hsa-miR-663b in IL21-mediated gene regulation. The box plots indicate the expression of *CCL17*, *DDR1*, *CD40* and *PIK3CD* genes in CLL cells from five different patients transfected with an irrelevant RNA sequence (indicated as irr) or with hsa-miR-663b (indicated as 663b). In addition, IL21-stimulated CLL cells were transfected with the irrelevant RNA (indicated as irr IL21) or with hsa-miR-663b antagonist (indicated as a663b IL21). Expression was tested by RT-qPCR. Statistical analysis was performed using the Kruskall—Wallis test.

RT-qPCR analyses showed that transfection with hsa-miR-663b down-regulated significantly the expression of *CCL17*, *CD40*, *DDR1*, *and PIK3CD* mRNAs in a similar fashion to IL21 (Fig 6). However, the hsa-miR-663b antagonist inhibited the down-regulation of these mRNAs in IL21-treated cells (Fig 6). As a control, we also tested the IL21-insensitive gene *CCL20*, which was not modulated by hsa-miR-663b or its antagonist (S4 Fig). These data indicated that IL21 modulated the expression of genes in CLL cells through mechanisms involving hsa-miR-663b and identified novel potential biological functions of this miRNA.

## Discussion

Our present findings indicate that IL21 can profoundly modify the mRNA and miRNA expression profiles in CD40-activated CLL cells. The gene and miRNA expression profiles were analyzed by microarray in a first cohort, and selected features were validated by qRT-PCR in the same case material and subsequently in an independent cohort. In addition, a coherent regulation of the same or orthologous genes was identified by submap analysis of a dataset of IL21-stimulated CLL cells [24] and three publicly available datasets of human and murine T cells stimulated by IL21 [41–43], suggesting a relevant role of the genes identified in our dataset. Finally, an integrated analysis of miRNA and mRNA expressions indicated that several miRNAs and mRNAs show significant anti-correlation, suggesting that IL21-modulated miRNAs regulate gene expression in CLL cells. Although we used IL21 at concentration of 80 ng/ml, which may represent a “pharmacological” dose, higher than physiological levels, our observations might be relevant considering potential therapeutic applications. In any case, the true physiological concentrations of IL21 in the local microenvironment of secondary lymphoid organs cannot be really established.

Genes regulated by IL21 functionally clustered into different modules. The first module (MEpink) contained genes involved in hematological system development, humoral immune response and immune disease. Indeed, this cluster comprised several genes encoding cytokine receptors, cytokines and the chemokines CCL17 and CCL22. The two most significant functional networks of genes within this cluster had, as highly connected nodes, NF-κB and TNF, respectively.

The NF-κB network included several down-regulated genes such as *NFKBIZ*, *NFKBIE* and *FCER*. *FCER* encodes FceR-II (CD23), which we reported previously as being down-regulated by IL21 [21]. However, within the same network, *STAT3* was up-regulated. IL21 induces STAT3 phosphorylation in CLL cells [21], and *MYC* is a known STAT3 target gene [45]. Accordingly, *MYC* and other STAT3-dependent genes [46], such as *STAT3*, *SOCS3*, *BCL3* and *BCL6*, were up-regulated by IL21 in CD40-activated CLL cells (S1 Table). Previous findings indicate that STAT3 up-regulates c-Myc expression in IL21-treated diffuse large B-cell lymphoma [30]. In this study, the STAT3/MYC pathway triggered apoptosis through the down-regulation of the anti-apoptotic Bcl-2 and Bcl-X(L) proteins. Here, we also found that IL21 inhibited the expression of the anti-apoptotic genes *BCL2* and *BCL2L1* (the latter encoding Bcl-X(L) protein) in CLL cells, whereas it up-regulated the pro-apoptotic genes *GZMB* and *BNIP3* (S1 Table), in agreement with a previous report [24].

In addition, a previous study reported that IL21 up-regulates granzyme-B expression in CLL and normal B-cells, resulting in the acquisition of cytotoxic properties and the induction of cell apoptosis [22]. In agreement with these data, *GZMB* was also up-regulated by IL21 in our study. Altogether, these data indicate that IL21 induces an imbalance in the expression of pro- and anti-apoptotic genes in CLL cells and suggest that IL21-mediated pro-apoptotic mechanisms are common to different B-cell neoplasms.

Interestingly, not only TNF, but also the TNF-related *LTB* gene, which encodes lymphotoxin-β, was down-regulated by IL21. Lymphotoxin-β is expressed highly in CLL cells and may support CLL cell proliferation *in vitro* [47], suggesting that IL21 may interfere with autocrine loops involving lymphotoxin-β in CLL cells.

The top functions of the second module (MEgreen) of IL21-modulated genes were RNA post-translational modification, cellular assembly and function, cell-to-cell signaling, cell movement and hematological system development. Pathway analysis showed PI3K and NF-κB complexes as highly connected nodes of two networks of regulated genes. Both PI3K and NF-κB are in biologically relevant pathways, which generate signals from several surface receptors, including the BCR complex and chemokine receptors, and mediate growth, survival and migration of CLL cells [48,49]. Importantly, we found that IL21 down-regulated the expression of the *PIK3CD* gene, which encodes PI3Kδ. This PI3K isoform is activated constitutively in CLL cells, most likely through stimuli arising from the microenvironment [48,49]. For this reason, PI3Kδ plays a crucial role in CLL biology and is considered a relevant target for therapy. Indeed, small molecule inhibitors of PI3Kδ, such as GS-1101, promote apoptosis of primary cells from CLL and other B-cell malignancies. In addition, CLL patients treated with GS-1101 showed a redistribution of CLL cells from the bone marrow and lymphoid organs in the periphery, and a reduction in CCL3 and CCL4 serum levels [49].

Importantly, in the present report, we showed that IL21 down-regulated the expression of the pro-inflammatory *CCL3*, *CCL4* and *CCL3L1* chemokine genes, and also those of *CCL17* and *CCL22*, which are Th2-related chemokine genes. These data were confirmed further in independent cohorts and collectively indicate that IL21 inhibits the production of chemokines that favor the crosstalk of CLL cells with supportive cells within the microenvironment [13–15]. In addition, IL21 up-regulated the expression of the Th1-related *CXCL9* and *CXCL10* chemokine genes, thus suggesting that IL21 mediates a shift from a Th2 to a Th1 chemokine profile in CLL cells. In murine tumor models, IL21 induces a Th1 immune response and anti-angiogenic effects mediated by CXCL9 and CXCL10 [50]. In general, most effects of IL21 on gene expression are similar in CLL and normal B-cells, indicating a role for these genes in normal B-cell responses. However, modulation of specific genes, such as *TNF*, *MYC*, *E2F1*, *EGR2* and *GAS-6*, occurred only in CLL B-cells, suggestive of a specific role of IL21 in CLL biology. In particular, the *EGR2* gene was recently found mutated in 8% of CLL cases with poor prognosis, suggesting a possible role of this gene in CLL pathogenesis, possibly through a deregulation of BCR intracellular signaling [51].

It is well known that specific miRNAs play an important role in CLL pathogeny and regulate the expression of several genes [52]. In addition, some cytokines were reported to regulate different miRNAs [32–33]. We therefore tested the hypothesis that specific sets of miRNAs could be modulated by IL21 and regulate gene expression in CLL cells. Indeed, our findings indicated that IL21 modulated significantly the expression of 63 miRNAs. To our knowledge, these miRNAs were not implicated previously in CLL biology, and some of them have yet unknown functions. However, the present data may underestimate the number of IL21-modulated miRNAs, as the miRBase 12 chip allowed the detection of only about 50% of the known miRNAs (miRBase 19). We then correlated miRNA to gene expression, using bioinformatic tools for data integration and prediction of potential miRNA/target gene interactions. The significant interactions involved 33 miRNAs, which were negatively correlated with 290 genes clustered in the different modules. Nine of these miRNAs appeared to regulate about 73% of expressed genes and could thus represent the main regulators of the effects of IL21 on CLL cells. In particular, we found that miR-125b-1*, miR-1228*, miR-296-3p and miR-1225-5p showed the largest number of potential interactions. Two of these miRNAs were ‘star’ sequences, which derive from pre-miRNA processing and were thought to be non-functional and rapidly degraded. However, this concept has been challenged, and several miR*, including miR-125b-1* and miR-1228*, have been renamed as hsa-miR-125b-1-3p and hsa-miR-1228-5p, respectively.

To confirm further that miRNAs modulated by IL21 can affect gene expression, we selected hsa-miR-663b. This miRNA is one of the miRNAs mostly up-regulated by IL21 and is also related to a single functional cluster of genes (that is, the MEpink module). In addition, hsa-miR-663b showed an inverse relationship with the expression of potentially relevant IL21-regulated genes, suggesting its possible involvement in some IL21 effects. Indeed, CD40L-activated CLL cells transfected with hsa-miR-663b showed a significant down-regulation of *CCL17*, *CD40*, *DDR1* and *PI3KCD* mRNAs. Conversely, the specific antagonist of hsa-miR-663b inhibited the down-regulation of the same genes mediated by IL21 treatment. Altogether, these data confirmed that IL21 can modulate the expression of genes in CLL cells through mechanisms involving the regulation of specific miRNAs and identify novel potential functions of hsa-miR-663b.

Although hsa-miR-663b regulated the expression of four mRNA targets and had potential binding sites in the 3′ UTR sequences, its activity might either be related to a direct action or involve indirect mechanisms through other targets. IL21 modulated hsa-miR-663b and its potential target genes not only in CLL, but also in normal B-cells, suggesting a general role of this miRNA in modulating both neoplastic and normal B-cell functions.

In conclusion, our data showed that IL21 is a potent modulator of gene expression in CLL cells. IL21 may regulate gene expression not only through specific transcription factors, such as STAT3, but also through the regulation of several miRNAs that potentially target more than 200 genes.

## Supporting Information

S1 Fig Subclass mapping analysis. Subclass mapping (SubMap) analysis comparing genome-wide molecular patterns identified in the current study (A1 and A2) and those identified in publicly available microarray datasets (B1 and B2). Red color indicates high confidence for correspondence; blue color indicates lack of correspondance. P values are indicated in the corresponding boxes. GSE50572: IL21-treated vs untreated human CLL cells [24]; GSE8685: IL21-treated vs untreated human Sezary cells [41]; GSE2059: IL21-treated vs untreated murine naive CD8+ T cells [42]; GSE19198: IL21-treated vs untreated murine pre-activated CD4+ T cells [43].
(PDF)

S2 Fig Ingenuity Pathway Analysis (IPA) of hsa-miR-663b targeted genes. The genes found as putative targets of hsa-miR-663b using MAGIA web-tool, identified a significant network (Infectious Disease, Cellular Movement, Hematological System Development and Function). Green indicates genes negatively correlated to hsa-miR-663b expression.
(PDF)

S3 Fig hsa-miR-663b expression in transfected and/or IL21-treated CLL cells. The box-plots indicate the relative expression of hsa-miR-663b in CLL cells from 5 different patients transfected with an irrelevant RNA sequence (irr) or with hsa-miR-663b (indicated as 663b). In addition, IL21-stimulated CLL cells were transfected with the irrelevant RNA (irr IL21) or with hsa-miR-663b antagonist (a663b IL21). Expression was tested by RT-qPCR. Statistical analysis was performed by Kruskall-Wallis test.
(PDF)

S4 Fig CCL20 expression in miRNA or antagomir-transfected CLL cells. CLL cells were transfected with an irrelevant RNA sequence (irr) or with hsa-miR-663b (663b). In addition, IL21-stimulated CLL cells were transfected with the irrelevant RNA (irr IL21) or with hsa-miR-663b antagonist (a663b IL21). Expression was tested by RT-qPCR. Statistical analysis was performed using the Kruskall—Wallis test.
(PDF)

S1 Table Genes differentially expressed between IL21-stimulated and paired control CCL cells. genes belonging to modules and their anti-correlation with differentially expressed miRNA.
(XLSX)

S2 Table List of the significant networks identified by IPA analysis in ME pink, ME green, and ME blue modules. The table summarizes the molecules present in each network (green up-regulated in CTR cells; red up-regulated in IL21-treated cells), the score (transformed from-logP, where P is calculated by the Fisher's exact test), the focus molecules, and the top functions.
(PDF)

S3 Table List of miRNAs differentially expressed between CLL cells stimulated with IL21 and controls. (XLSX)

S4 Table miRNA validation. (PDF)

S5 Table miRNAs potentially involved in expression regulation of genes belonging to identified modules. (PDF)
