# Supplementary figures and images for: Interleukin 21 Controls mRNA and MicroRNA Expression in CD40-Activated Chronic Lymphocytic Leukemia Cells
Source: PLoS One. 2015 Aug 25;10(8):e0134706. doi: 10.1371/journal.pone.0134706 (PMC4549109; doi:10.1371/journal.pone.0134706)

S1 Fig

GSE50572

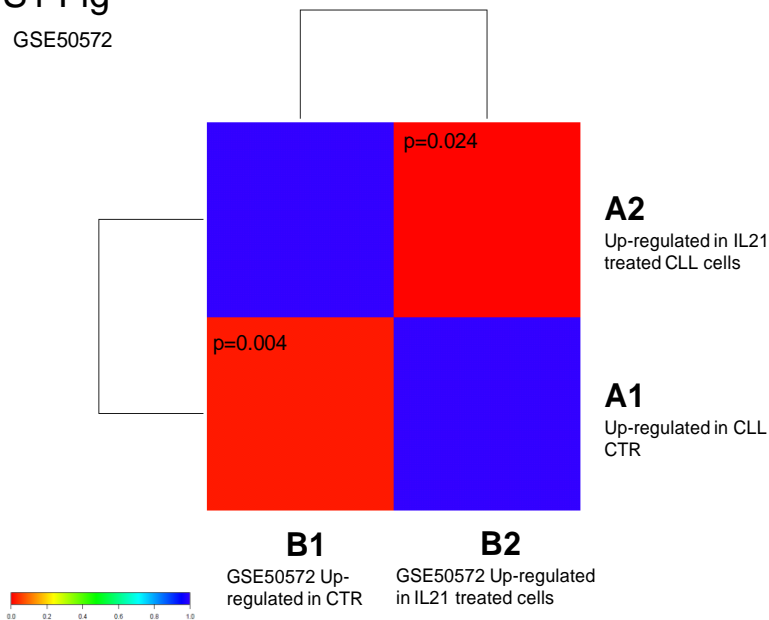

GSE8685

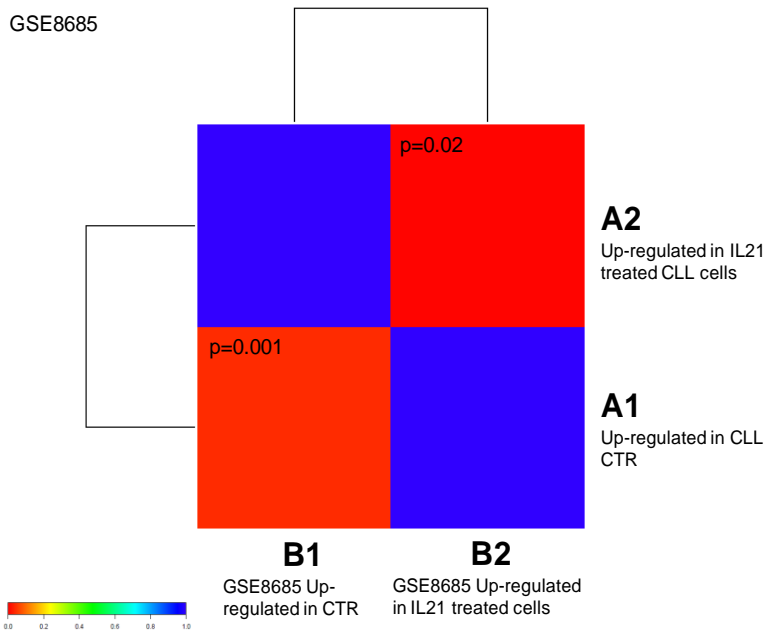

GSE2059

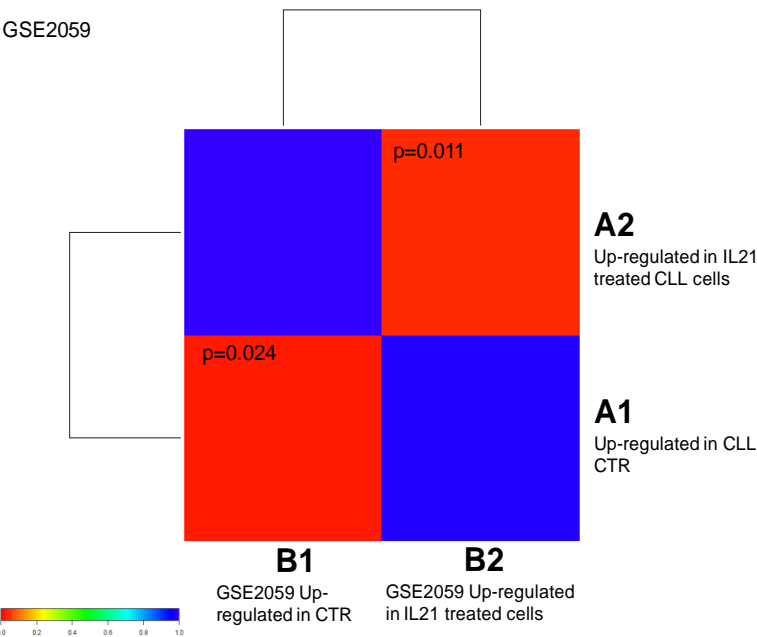

GSE19198

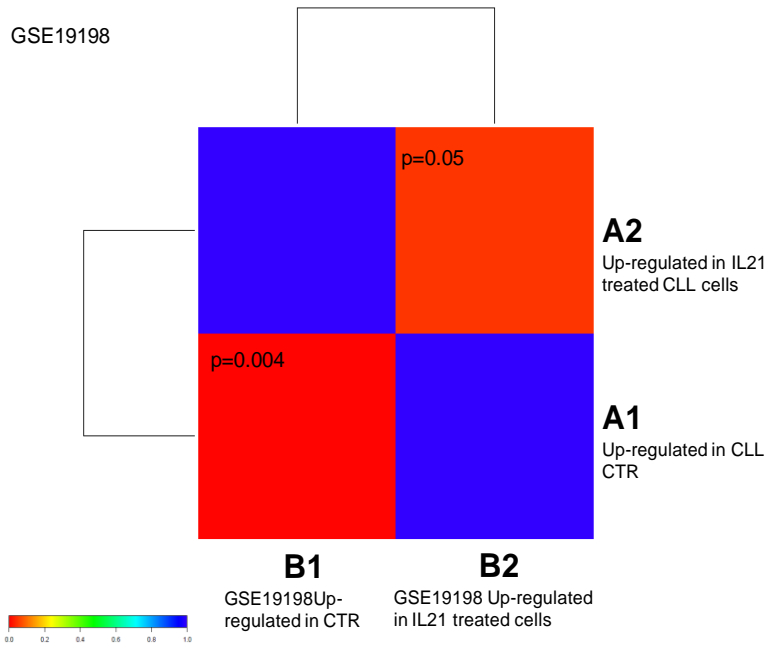

Supplement: S1 Fig — Subclass mapping (SubMap) analysis comparing genome-wide molecular patterns identified in the current study (A1 and A2) and those identified in publicly available microarray datasets (B1 and B2). Red color indicates high confidence for correspondence; blue color indicates lack of correspondance. P values are indicated in the corresponding boxes. GSE50572: IL21-treated vs untreated human CLL cells [24]; GSE8685: IL21-treated vs untreated human Sezary cells [41]; GSE2059: IL21-treated vs untreated murine naive CD8+ T cells [42]; GSE19198: IL21-treated vs untreated murine pre-activated CD4+ T cells [43]. (PDF) [file pone.0134706.s001.pdf]

S2 Fig

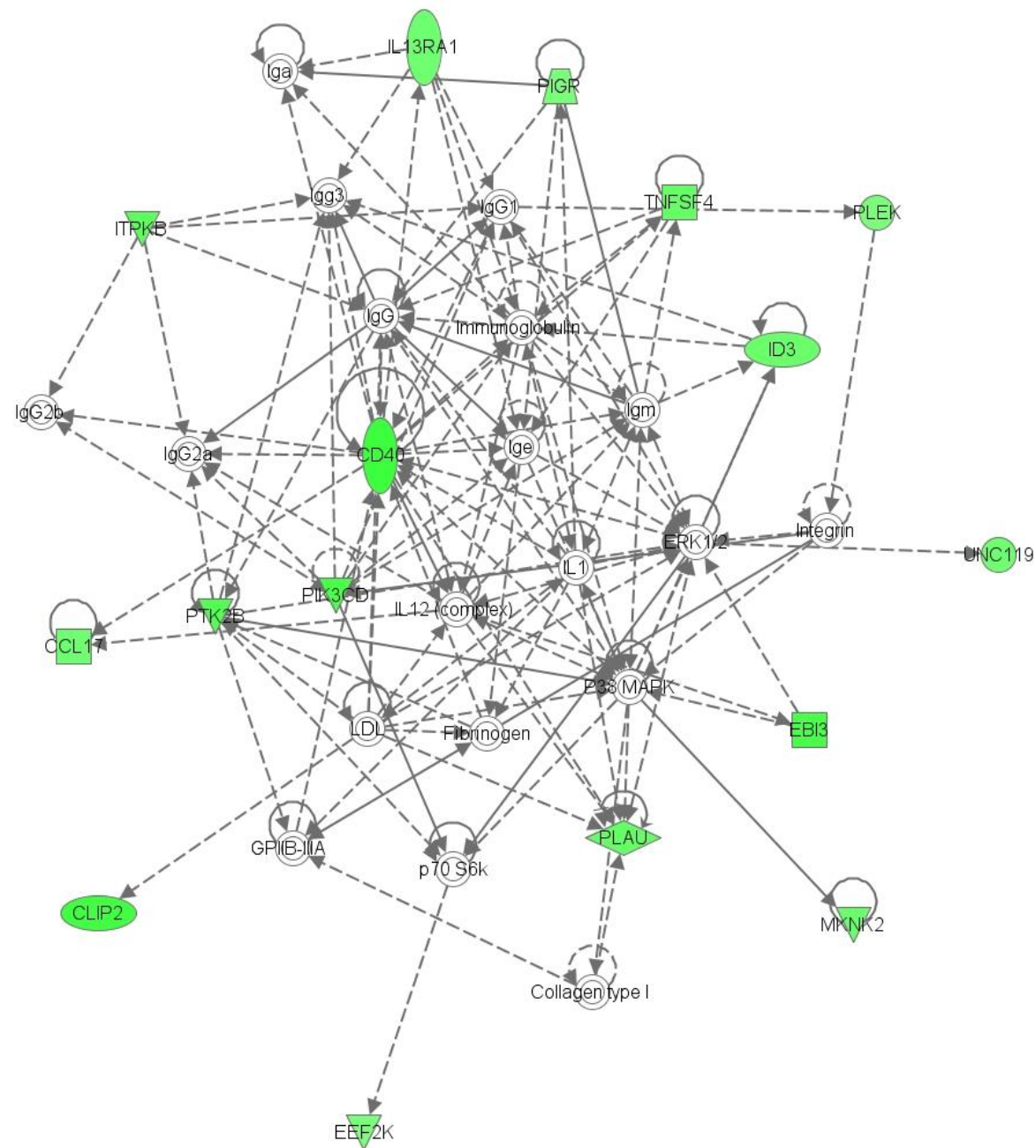

Supplement: S2 Fig — The genes found as putative targets of hsa-miR-663b using MAGIA web-tool, identified a significant network (Infectious Disease, Cellular Movement, Hematological System Development and Function). Green indicates genes negatively correlated to hsa-miR-663b expression. (PDF) [file pone.0134706.s002.pdf]

S3 Fig

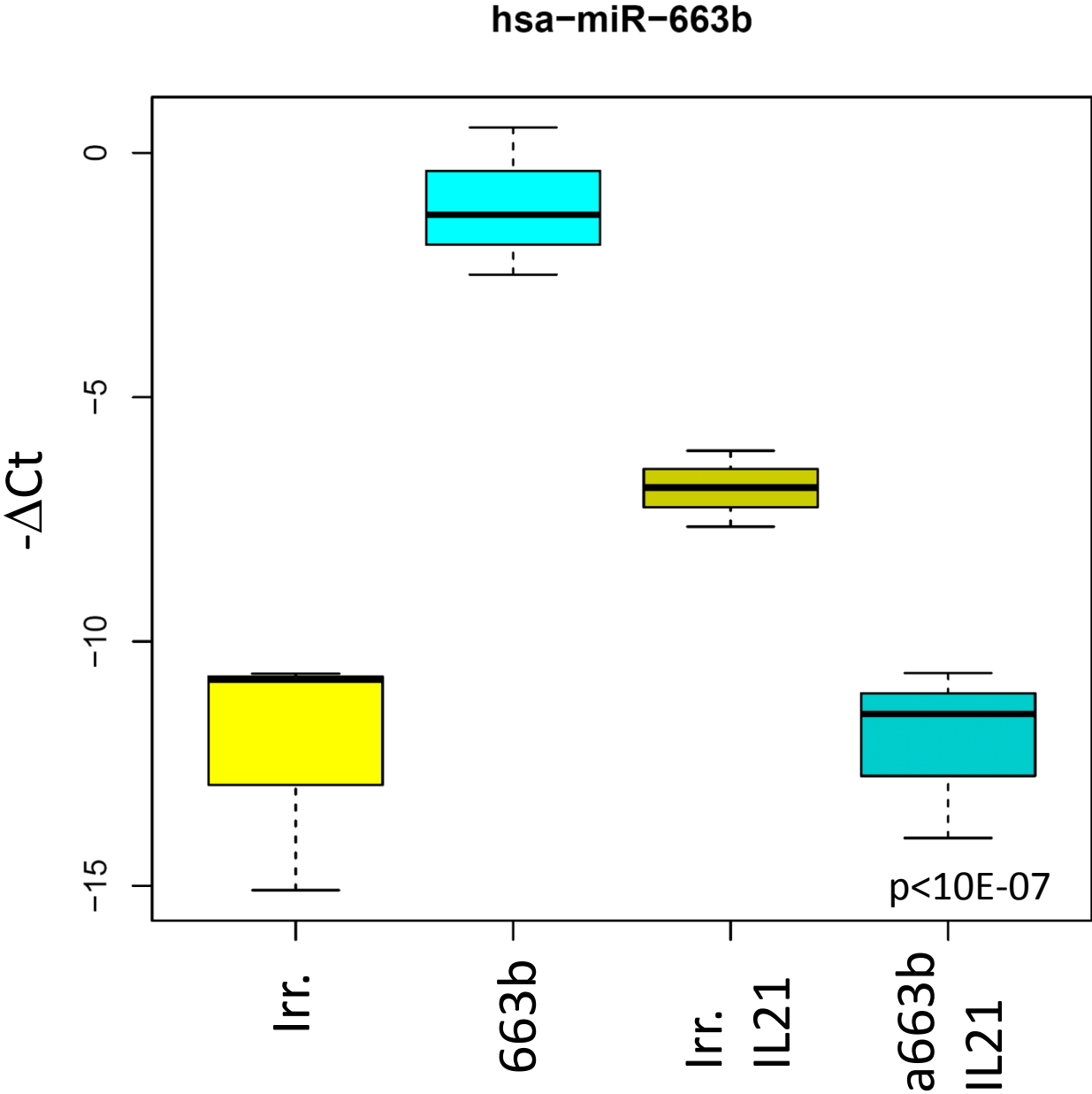

Supplement: S3 Fig — The box-plots indicate the relative expression of hsa-miR-663b in CLL cells from 5 different patients transfected with an irrelevant RNA sequence (irr) or with hsa-miR-663b (indicated as 663b). In addition, IL21-stimulated CLL cells were transfected with the irrelevant RNA (irr IL21) or with hsa-miR-663b antagonist (a663b IL21). Expression was tested by RT-qPCR. Statistical analysis was performed by Kruskall-Wallis test. (PDF) [file pone.0134706.s003.pdf]

S4 Fig

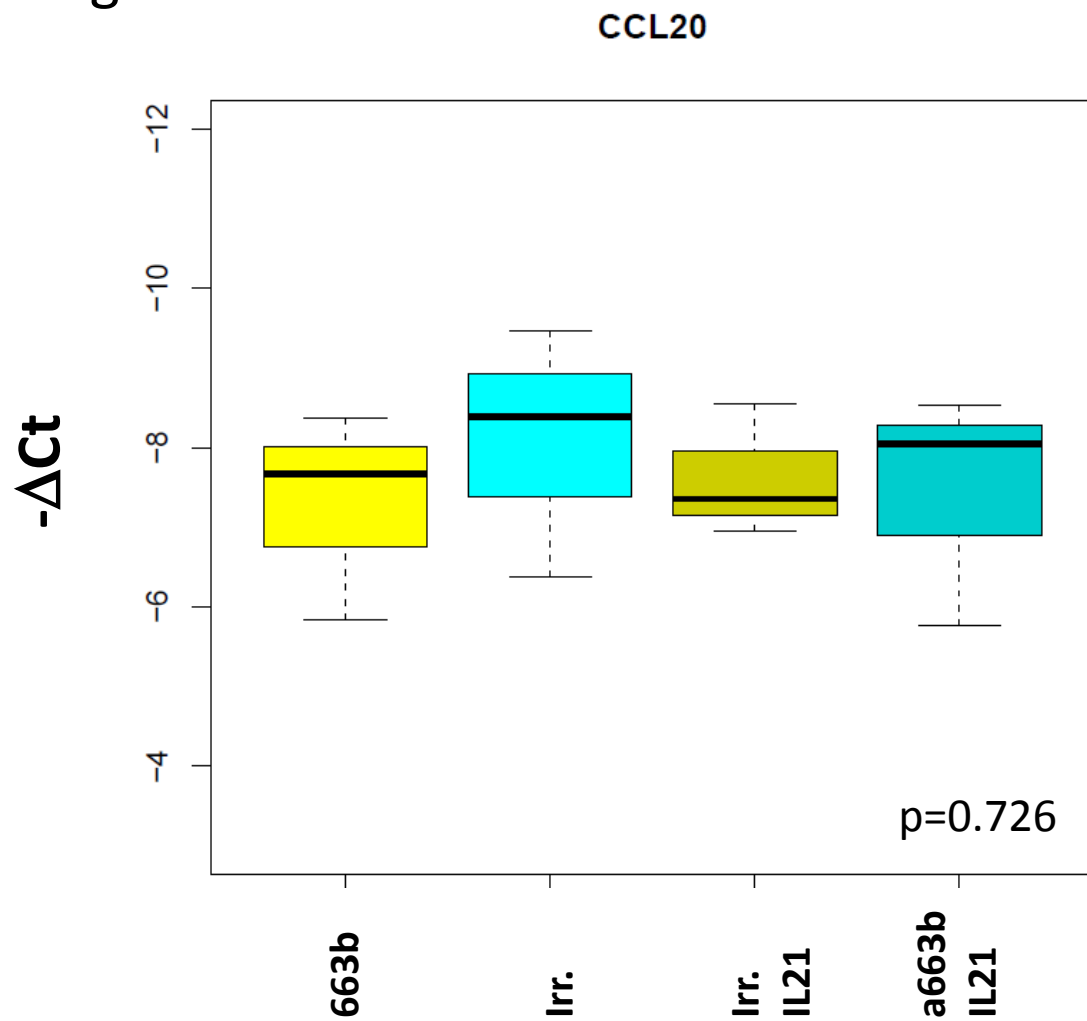

Supplement: S4 Fig — CLL cells were transfected with an irrelevant RNA sequence (irr) or with hsa-miR-663b (663b). In addition, IL21-stimulated CLL cells were transfected with the irrelevant RNA (irr IL21) or with hsa-miR-663b antagonist (a663b IL21). Expression was tested by RT-qPCR. Statistical analysis was performed using the Kruskall—Wallis test. (PDF) [file pone.0134706.s004.pdf]
