# Supplementary material for: Interleukin 21 Controls mRNA and MicroRNA Expression in CD40-Activated Chronic Lymphocytic Leukemia Cells
Source: PLoS One. 2015 Aug 25;10(8):e0134706. doi: 10.1371/journal.pone.0134706 (PMC4549109; doi:10.1371/journal.pone.0134706)
Supplement: S2 Table — The table summarizes the molecules present in each network (green up-regulated in CTR cells; red up-regulated in IL21-treated cells), the score (transformed from-logP, where P is calculated by the Fisher's exact test), the focus molecules, and the top functions. (PDF) [file pone.0134706.s006.pdf]

S2 Table

ID	Molecules in Network	Score	Focus Molecules	Top Functions
1	ADAM8, Alpha Actinin, Alpha catenin, AMPD3, ARHGAP17, Cadherin, CDC42EP4, DNASE1L3, Erm,F Actin, FNBP1, Fructose 2,6 Bisphosphatase, GNE, HCN3, HERC1, Il8r, KMO, MARCH1, MARCKS, MARCKSL1, Mlc, OAS3, Pak, PFKFB3, PFKL, Rho gdi, RHOG, Rock, SNN, SRGN, ST3GAL5, TNF, TST, Ube3, UBQLN2	36	23	Lipid Metabolism, Small Molecule Biochemistry, Hematological System Development and Function
2	ALCAM, BATF, CLIP2, DUSP5, Fascin, FCER2, glutathione peroxidase, GPX4, Hat, HERC5, ICOSLG, IgG2b, IgG2c, Ikk (family), Il12 receptor, ITPKB, Mhc class ii, MUC1, NFkB (complex), NFkB (family), NfkB-RelA, NfkB1-RelA, NFKBID, NFKBIE, NFKBIZ, peptidase, PRDX2, PTPN2, RHOH, RNF19B, SLC2A6, sphingomyelinase, Stat3-Stat3, TNFRSF13B, VMP1	33	20	Humoral Immune Response, Protein Synthesis, Cellular Development
3	Adaptor protein 1, ALDH2, CD58, CD83, DTX3L, EBI3, ERK1/2, FCGR1A/2A/3A, HLA-DOA, HLA-DOB, HLA-DPB1, IFN alpha/beta, IFN TYPE 1, Ifnar, Iga, Igf, IL12A, IL13RA1, KLF13, LIMS1, MHC,MHC Class I (complex), MHC Class II (complex), MMD, PARP9, PTPase, Rab5, RUFY3, SH2B3, STAT3/5, Tlr, TLR2/3/4/9, TYK2, UCP2, UNC119	31	19	Endocrine System Disorders, Gastrointestinal Disease, Immunological Disease
1	ACP5, Ap1, BATF3, CAPG, CD68, CEACAM1 (includes others), CLNK, Collagen type I, Collagen(s), Ctbp, CTH, DDX21, EBNA1BP2, FUT7, GNL3, HMBS, HNRNPAB, LDL, LYAR, Mek, MTHFD2, NAGK, NHP2, NOLC1, NOP56, NR4A2, Pdgf (complex), PDGF BB, PI3K (complex), PSAT1, PUS7, RANBP1, RCC1, STAT5a/b, Tgf beta	46	24	RNA Post-Transcriptional Modification, Cellular Assembly and Organization, Cellular Function and Maintenance
2	60S ribosomal subunit, BOP1, Calmodulin, CD3, Ck2, Creb, ERK, estrogen receptor, Hdac, HEATR3, HISTONE, Hsp90, HUWE1, IFRD2, LY9, MYC, NETO2, NME1 (includes EG:18102), ODC1, Pde, PDE4B, PDE7A, PNP,PPA1, Ribosomal 40s subunit, RNA polymerase I, Rnr, RPL6, RPL29 (includes EG:367874), RPS7, RPS15, STOM, VAMP1, WARS, WDR12	36	21	RNA Post-Transcriptional Modification, Infectious Disease, Cardiovascular Disease
3	ABCG1, CCL3, CCL4, CCL3L1/CCL3L3, CD69, CXCL9, CXCL10, HLA-DMB, HLA-DR, HRSP12, IFIT2, Ifn, IFN alpha/beta, IFN Beta, Ifn gamma, IFN TYPE 1, IgG2a, IL10RA, IL12 (complex), IL28RA, LTB, lymphotoxin, MIP1, NCOR-LXR-Oxysterol-RXR-9 cis RA, NFkB (complex), Nr1h, PECAM1, PI3K (family), SLC2A5, SLC7A1, ST3GAL1, Tlr, Tnf, TYROBP, WNT10A	33	19	Cell-To-Cell Signaling and Interaction, Cellular Movement, Hematological System Development and Function
4	ACACA, ACAT1, AK1, Akt, ALOX5AP, AMPK, C1QBP, Cyclin A, DDIT4, E2f, ENO1, EXOSC5, FASN, FSH, Growth hormone, hCG, hexokinase, HK2, Insulin, LDHA, Lh, MAP2K1/2, MIF, MTORC1, PA2G4, PAICS, PP2A, PPAT, PRKAA, RAPGEF3, Rb, SH3PXD2A, SLC29A1, STRA13, T3-TR-RXR	32	19	Carbohydrate Metabolism, Lipid Metabolism, Nucleic Acid Metabolism
1	APC (complex), CDC45, CDC25A, CDT1, CHEK1, CKS2, Collagen Alpha1, Cyclin A, Cyclin B, DTL, DUT, E2F1, E2f, FEN1, GAS6, HAMP, HNRNPA1, LGALS1, MAD2L1, MCM4, MCM5, MCM6, MCM7, Mcm, MCM10 (includes EG:307126), NCAPG, NFkB (complex), ORC1 (includes EG:18392), ORC6 (includes EG:23594), PTP4A3,Rb, SLC25A10, TIMP2 (includes EG:21858), TSC22D1, UBE2C	66	27	DNA Replication, Recombination, and Repair, Cell Cycle, Cancer
2	ADAM15,Akt, BUB1 (includes EG:100307076), CASP7, caspase, CD3, CDCA5, CDCA7, CKS1B, DLK1, DSCC1, DUSP3, EGR2, ERK,ERK1/2, Focal adhesion kinase, FSH,hCG, Histone h3, HMMR, Igm, Insulin, Jnk, KISS1R, Lh, LPIN1, Mek, NME4, P38 MAPK, PITPNA, Pkc(s), SCARB1, TRIP13, UHRF1, Vegf	38	18	Cancer, Lipid Metabolism, Molecular Transport
3	ABCA9, ACOT7, ARID4A, ARIH2, ARPC4, ARPC5L, C16orf59, CENPN, CLIP3, DEDD, DOCK7, EXO1 (includes EG:26909), FANCF, FANCL, FEN1, FEZ2, GINS2, GINS3, GINS4, GPSM1, IMPDH1, KPNA4, LNX2, LRRC20, MARCH5, MGRN1, MSH3 (includes EG:17686), PNMA2, RECQL, RENBP, RNF144B, RPP14, UBC, UBE2T, ZBED1	31	15	Cellular Assembly and Organization, Cellular Function and Maintenance, Post-Translational Modification
