# Supplementary material for: Interleukin 21 Controls mRNA and MicroRNA Expression in CD40-Activated Chronic Lymphocytic Leukemia Cells
Source: PLoS One. 2015 Aug 25;10(8):e0134706. doi: 10.1371/journal.pone.0134706 (PMC4549109; doi:10.1371/journal.pone.0134706)
Supplement: S4 Table — (PDF) [file pone.0134706.s008.pdf]

S4 Table: miRNA validation

	Arrays	Technical validation by RT-qPCR		Technical validation by RT-qPCR		Normal B cells RT-qPCR	
**miRNA ID**	**Fold change***	**Fold change***	**Parametric p-value**	**Fold change***	**Parametric p-value**	**Fold change***	**Parametric p-value**
**hsa-miR-663b**	1.47	3.02	0.00357	2.26	0.000941	1.42	0.0077
**hsa-miR-125b-1***	0.73	0.36	0.00405	0.42	0.00703	0.93	0.202
**hsa-miR-708**	0.70	0.43	0.0352	0.48	0.00936	1.11	0.588

***IL21/CTR**
