# Supplementary material for: Interleukin 21 Controls mRNA and MicroRNA Expression in CD40-Activated Chronic Lymphocytic Leukemia Cells
Source: PLoS One. 2015 Aug 25;10(8):e0134706. doi: 10.1371/journal.pone.0134706 (PMC4549109; doi:10.1371/journal.pone.0134706)
Supplement: S5 Table — (PDF) [file pone.0134706.s009.pdf]

**S5 Table:** miRNAs potentially involved in expression regulation of genes belonging to identified modules

| miRNA ID | No. genes anti-correlated | modules | | |
|---|---|---|---|---|
| | | ME pink | MEgreen | MEblue |
| hsa-miR-296-3p | **64** | 47 | 12 | 5 |
| hsa-miR-125b-1* | **51** | 21 | 18 | 12 |
| hsa-miR-1225-5p | **35** | 14 | 19 | 2 |
| hsa-miR-1228* | **25** | 16 | 6 | 3 |
| hsa-miR-326 | **11** | 3 | 5 | 3 |
| hsa-miR-92a-1* | **10** | 7 | 2 | 1 |
| hsa-miR-1205 | **6** | 1 | 4 | 1 |
| hsa-miR-130a | **5** | 2 | 2 | 1 |
| hsa-miR-381 | **5** | 2 | 2 | 1 |
| hsa-miR-663b | **28** | 27 | 1 | - |
| hsa-miR-92a | **6** | 6 | - | - |
| hsa-miR-24 | **5** | 4 | 1 | - |
| hsa-miR-22 | **4** | - | 3 | 1 |
| hsa-miR-657 | **3** | 3 | - | - |
| hsa-miR-922 | **3** | 2 | - | 1 |
| hsa-miR-943 | **3** | 3 | - | - |
| hsa-miR-1204 | **3** | 2 | 1 | - |
| hsa-miR-7 | **2** | - | - | 2 |
| hsa-miR-25 | **2** | 2 | - | - |
| hsa-miR-193b | **2** | 1 | 1 | - |
| hsa-miR-652 | **2** | - | 2 | - |
| hsa-miR-708 | **2** | - | 2 | - |
| hsa-miR-874 | **2** | 1 | 1 | - |
| hsa-miR-1270 | **2** | 2 | - | - |
| hsa-miR-101 | **1** | 1 | - | - |
| hsa-miR-107 | **1** | 1 | - | - |
| hsa-miR-181c | **1** | 1 | - | - |
| hsa-miR-193a-5p | **1** | - | 1 | - |
| hsa-miR-331 | **1** | - | 1 | - |
| hsa-miR-542-5p | **1** | 1 | - | - |
| hsa-miR-646 | **1** | - | - | 1 |
| hsa-miR-720 | **1** | - | 1 | - |
| hsa-miR-1323 | **1** | 1 | - | - |
| **Total** | **290** | **171** | **85** | **34** |

**(cut-off -0.3 correlation)**
